# Novel Lipolytic Enzymes Identified from Metagenomic Library of Deep-Sea Sediment

**DOI:** 10.1155/2011/271419

**Published:** 2011-08-07

**Authors:** Jeong Ho Jeon, Jun Tae Kim, Hyun Sook Lee, Sang-Jin Kim, Sung Gyun Kang, Sang Ho Choi, Jung-Hyun Lee

**Affiliations:** ^1^Marine Biotechnology Research Center, Korea Ocean Research & Development Institute, P.O. Box 29, Ansan 425-600, Republic of Korea; ^2^National Research Laboratory of Molecular Microbiology and Toxicology, Department of Agricultural Biotechnology, Seoul National University, Seoul 151-921, Republic of Korea; ^3^Department of Marine Biotechnology, University of Science and Technology, Daejeon 305-333, Republic of Korea

## Abstract

Metagenomic library was constructed from a deep-sea sediment sample and screened for lipolytic activity. Open-reading frames of six positive clones showed only 33–58% amino acid identities to the known proteins. One of them was assigned to a new group while others were grouped into Families I and V or EstD Family. By employing a combination of approaches such as removing the signal sequence, coexpression of chaperone genes, and low temperature induction, we obtained five soluble recombinant proteins in *Escherichia coli*. The purified enzymes had optimum temperatures of 30–35°C and the cold-activity property. Among them, one enzyme showed lipase activity by preferentially hydrolyzing *p*-nitrophenyl palmitate and *p*-nitrophenyl stearate and high salt resistance with up to 4 M NaCl. Our research demonstrates the feasibility of developing novel lipolytic enzymes from marine environments by the combination of functional metagenomic approach and protein expression technology.

## 1. Introduction

Lipolytic enzymes such as esterases and lipases belong to the class of carboxylic ester hydrolases that catalyze both the hydrolysis and synthesis of ester bonds. Lipolytic enzymes have been classified into eight families based on the conserved sequence motifs and biological properties [1]. They share a characteristic *α*/*β* hydrolase fold in the three-dimensional structure, but show differences in substrate preferences. Esterases (EC 3.1.1.1) hydrolyze water-soluble or emulsified esters with short-chain carboxylic acids (≤10 carbon atoms), while lipases (EC 3.1.1.3) prefer long-chain acylglycerides (≥10 carbon atoms) [2]. Esterases and lipases have a wide range of biotechnological applications, such as organic chemical processing, detergent formulation, synthesis of biosurfactants, the oleochemical industry, the dairy industry, the agrochemical industry, paper manufacturing, nutrition, cosmetics, and pharmaceutical processing [3–6]. Therefore, the identification of novel esterases/lipases will be a useful tactic for finding novel biocatalysts. 

The metagenomic approach, direct cloning of genomes of all microorganisms present in a given habitat, can be accessing the potential of nonculturable microorganisms [7–10]. In detail, metagenomic libraries were constructed from DNA of diverse environmental samples in cloning vectors including cosmid, fosmid and bacterial artificial chromosome (BAC) [11–13] and host strains. Two major strategies have been pursued to identify novel biocatalysts or genes with new functions for biotechnological applications. The first approach uses function-based screenings of metagenomic DNA libraries, and the second one includes sequence-based searches [14–16]. Through functional screens of metagenomic libraries, several genes encoding lipolytic enzymes have been previously identified from various environmental samples [17–21].

We have been applying metagenomic approach to search for new lipolytic enzymes from marine environmental samples such as deep-sea and arctic seashore sediments, which possess untouched and potential resources [22–24]. Several novel esterases/lipases have been identified with unique properties including cold activity. In this study, we used another sediment core sample of deep sea which had been explored in our previous researches and could unveil the existence of novel lipolytic enzymes. Here, we describe the identification of novel lipolytic enzyme-encoding genes from metagenomic library, the enhancement of soluble protein expression, and the biochemical characterization of the purified enzymes.

## 2. Materials and Methods

### 2.1. Strains, Library Construction, and Screening Escherichia coli

DH5*α* (Stratagene, La Jolla, CA, USA), EPI300-T1R (Epicentre, Madison, WI, USA), and BL21(DE3) (Novagen, Madison, WI, USA) were used as host strains for cloning and expression. pBluescript SK- (Stratagene), pET-24a(+) vector (Novagen), and fosmid vector (Epicentre) were used as vectors.

### 2.2. Metagenomic Library Construction and Screening for Lipolytic Clones

Deep-sea sediment sample was collected from the southern clam beds area around the summit of Edison Seamount in the New Ireland Fore-arc near Papua New Guinea (3°89′S/152°49′E; depth 1,440 m). DNA from the sediment sample was extracted based on a previously described method [25] with minor modifications. After extraction, the DNA was further purified by gel electrophoresis in a 1% low-melting-temperature agarose gel (FMC Bioproducts, Rockland, ME) containing 1% polyvinyl polypyrrolidone (Sigma, St. Louis, MO). Gel electrophoresis was performed at 35 V for 13 h, and DNA fragments of approximately 40 to 50 kb were then isolated from the gel. The isolated DNA was end-repaired with End-It DNA End-Repair kit (Epicentre, Madison, WI), which caused the DNA to be blunt ended and 5′-phosphorylated. The blunt-ended DNA was ligated into a pCC1FOS vector (Epicentre, Madison, WI). Lambda packaging extracts were added to ligations, and infection of phage T1-resistant EPI300-T1^R^ cells was performed according to the manufacturer's instructions. The *E. coli* transformants were transferred to 96-well microtiter plates and stored at −80°C. To screen for esterase/lipase activity, the transformants were plated on Luria-Bertani (LB) agar plates containing 12.5 **μ**g*/*mL of chloramphenicol and 1% tributyrin as a substrate. Colonies were incubated for one day at 37°C and subsequently incubated for a week at 4°C. Candidates surrounded by a clear halo on the plate were selected. The positive clones were reconfirmed and subcloned.

Fosmid clones showing lipolytic activity on the tributyrin agar plate were inoculated into 200 mL of LB broth containing 12.5 **μ**g*/*mL of chloramphenicol. After overnight incubation at 37°C, the cells were harvested by centrifugation at 5,000 ×g for 15 min and washed twice with distilled water. The fosmid DNA was purified using the alkaline lysis method [26] with minor modifications and was randomly sheared by nebulization according to the manufacturer's instruction (Invitrogen, Carlsbad, CA). After nebulization, DNA fragments of 2 to 4 kb were isolated from a 0.6% low-melting-temperature agarose (FMC Bioproducts, Rockland, ME) gel and end-repaired to generate blunt ends. The blunt-ended DNA was ligated into the *Eco*RV site of pBluescript SK(−) (Stratagene, La Jolla, CA), and the ligations were introduced into* E. coli* DH5*α* cells. The *E. coli* transformants were plated onto LB agar plates containing 100 **μ**g*/*mL of ampicillin and 1% tributyrin. After incubation at 37°C for 24 h, colonies surrounded by a clear halo were selected. Nucleotide sequencing was performed with the automated sequencer (ABI3100) using the BigDye terminator kit (PE Applied Biosystems, Foster City, CA). The DNA sequence was determined by primer walking in both directions and assembled using the ContigExpress program of the Vector NTI suite 7 software package (InforMax, North Bethesda, Md.). The open reading frame (ORF) was detected using the ORF search tool provided by the National Center for Biotechnology Information (NCBI). Sequence homology searches were performed with the basic local alignment search tool (BLAST) program [27]. Signal sequence search was performed with the SignalP 3.0 program [28]. Multiple alignments between protein sequences were performed with the ClustalW program [29]. The phylogenetic tree was constructed by the neighbor-joining method [30] using the Molecular Evolutionary Genetics Analysis 4.1 software (MEGA, version 4.1) [31].

### 2.3. Overexpression and Purification of the Lipolytic Enzyme-Encoding Genes

The lipolytic enzyme genes were amplified by PCR with primer pairs, and the amplified DNA fragments were inserted into the pET-24a(*+*) expression vector (Table 1). Three recombinant plasmids including genes of EM3L1, EM3L2, and EM3L3 were transformed into *E. coli* BL21 (DE3) cells while three recombinant plasmids including genes of EM3L4, EM3L6, and EM3L7 were transformed into *E. coli* BL21 (DE3) expressing molecular chaperones GroEL-GroES with pGro7 (Takara, Kyoto, Japan) and the transformants were inoculated into LB medium containing 20 **μ**g/mL of chloramphenicol and 50 **μ**g/mL of kanamycin for plasmid selection and 0.5 mg/mL L-arabinose for induction of chaperone expression. A transformants were cultivated at 37°C, and 1 mM isopropyl-*β*-D-thiogalactopyranoside (IPTG) was added to induce gene expression when the optical density at 600 nm reached 0.4. After incubation for 16 h at 16°C, the cells were harvested by centrifugation (6,000 ×g, 20 min, 4°C) and resuspended in 50 mM Tris-HCl buffer (pH 8.0) containing 0.1 M KCl and 10% glycerol. The cells were disrupted by sonication and centrifuged (20,000 ×g, 1 h, 4°C). The resulting supernatants were applied to a column of TALON metal-affinity resin (BD Biosciences Clontech, Palo Alto, CA) and washed with 10 mM imidazole (Sigma, St. Louis, MO) in 50 mM Tris-HCl buffer (pH 8.0) containing 0.1 M KCl and 10% glycerol, and the enzymes were eluted with 300 mM imidazole in the buffer. The protein concentration was measured by the Bradford method using the Bio-Rad protein assay kit (Bio-Rad, Hercules, CA) with bovine serum albumin as a standard [32]. The purity of the protein was examined by sodium dodecyl sulfate-polyacrylamide gel electrophoresis (SDS-PAGE) under denaturing conditions as described method [33].

### 2.4. Characterization of Lipolytic Enzymes

Esterase and lipase activities were measured by a spectrophotometric method using *p*-nitrophenyl butyrate and *p*-nitrophenyl palmitate (Sigma, St. Louis, MO) as the substrate, respectively. The reaction mixture contained *p*-nitrophenyl butyrate in acetonitrile, Tris-HCl buffer, and the enzyme solution. *p*-nitrophenyl palmitate solutions were mixed with Tris-HCl buffer containing Triton X-100 as emulsifying agent. After incubation at each optimum temperature for 5 min, the absorbance at 405 nm was measured to detect the released *p*-nitrophenol. One unit of esterase and lipase activity was defined as the amount of enzyme required to release 1 **μ**mol of *p*-nitrophenol from *p*-nitrophenyl butyrate or *p*-nitrophenyl palmitate per min. 

The optimum temperature of the enzymes was determined at various temperatures of 5 to 65°C. The optimum pH was determined over a pH range of 4.0 to 10.0, using the following buffer systems: 50 mM sodium acetate (pH 4.0 to 6.0), 50 mM sodium phosphate (pH 6.0 to 7.5), 50 mM Tris-HCl (pH 7.5 to 8.5), and 50 mM CHES (pH 8.5 to 10.0). The substrate specificity was determined with different aliphatic side chain, C2 (acetate), C4 (butyrate), C6 (hexanoate), C8 (octanoate), C10 (decanoate), C12 (laurate), C14 (myristate), C16 (palmitate), and C18 (stearate) as substrates.

Various metal ions (MnCl_2_, MgCl_2_, CaCl_2_, CuCl_2_, ZnSO_4_, FeSO_4_, CoSO_4_, and NiSO_4_) and enzyme inhibitors, Phenylmethylsulfonyl fluoride (PMSF) and Ethylenediaminetetraacetic acid (EDTA), at a final concentration of 1 mM were added to the enzyme in 50 mM Tris-HCl buffer (pH 7.5) then assayed for enzyme activity after preincubation at 35°C for 1 hr. The effects of detergents on enzyme activity were determined by incubating the enzyme in 50 mM Tris-HCl buffer (pH 7.5) containing 1% (w/v) of the detergents SDS, Triton X-100, and Tween-20, -40, -60, -80 at 35°C for 1 hr.

The effect of NaCl concentration on enzyme activity was measured using *p*-nitrophenyl hexanoate as the substrate. The reaction mixture contained enzyme solution in 50 mM Tris-HCl (pH 7.5) containing different final NaCl concentrations ranging from 0.5 to 4 M. The mixture was preincubated at 35°C for 30 min, and then the enzyme activity was detected by adding *p*-nitrophenyl hexanoate.

### 2.5. Nucleotide Sequence Accession Numbers

The obtained nucleotide sequences have been deposited in the GenBank database under the accession numbers EM3L1 (GQ340923), EM3L2 (GQ340924), EM3L3 (GQ340925), EM3L4 (GQ340926), EM3L6 (GQ340927), and EM3L7 (GQ340928).

## 3. Results

### 3.1. Screening and Primary Sequence Analysis of Lipolytic Enzyme-Encoding Genes

To explore the untapped esterases/lipases in marine environment, metagenomic approach was applied to the samples from deep-sea sediment, located near a small volcanic cone named the Edison Seamount at a depth of 1440 m where there are extensive clam beds associated with the low-temperature vents [34]. Deep-sea sample was chosen to represent the psychrophilic environment with average temperature below 4°C. A metagenomic library consisting of 81,100 fosmid clones was constructed in a fosmid vector, pCC1FOS. Each clone contained an insert of approximately 15 to 33 kb. Fosmid clones having lipolytic activity were identified by activity screening on agar plates containing 1% tributyrin (TBN). As a result, 6 positive clones (designated as pFosEM3L1, pFosEM3L2, pFosEM3L3, pFosEM3L4, pFosEM3L6, and pFosEM3L7) were obtained by zones of clearance around the colonies.

The 6 fosmid clones were subjected to further subcloning into the pBluescript SK(−) plasmid. The subclones with lipolytic activity were selected on TBN plates again. Sequence analyses of the 2-3 kb DNA inserts in the subclones revealed the presence of ORFs encoding putative *α*/*β* hydrolases with G + C contents of 46.5–66.6%. A BLAST search of the amino acid sequences of six ORFs indicated that they yielded identities of less than 60% to proteins in the database. The deduced amino acid sequences of EM3L1, EM3L2, EM3L3, EM3L4, EM3L6, and EM3L7 showed the highest similarity to *α*/*β* hydrolase fold protein (YP_003395264) from *Conexibacter woesei* DSM 14684 (39% identity), hypothetical protein (ZP_01915829) from *Limnobacter* sp. MED105 (49% identity), triacylglycerol acyl hydrolase (AAK07450) from *Moritella marina* (49% identity), LpqC (ZP_01463024) from *Stigmatella aurantiaca* DW4/3-1 (39% identity), *α*/*β* hydrolase fold protein (ZP_01697334) from *Bacillus coagulans* 36D1 (33% identity), and lipase (ACJ13070) from uncultured bacterium (58% identity), respectively.

**Figure 1:**
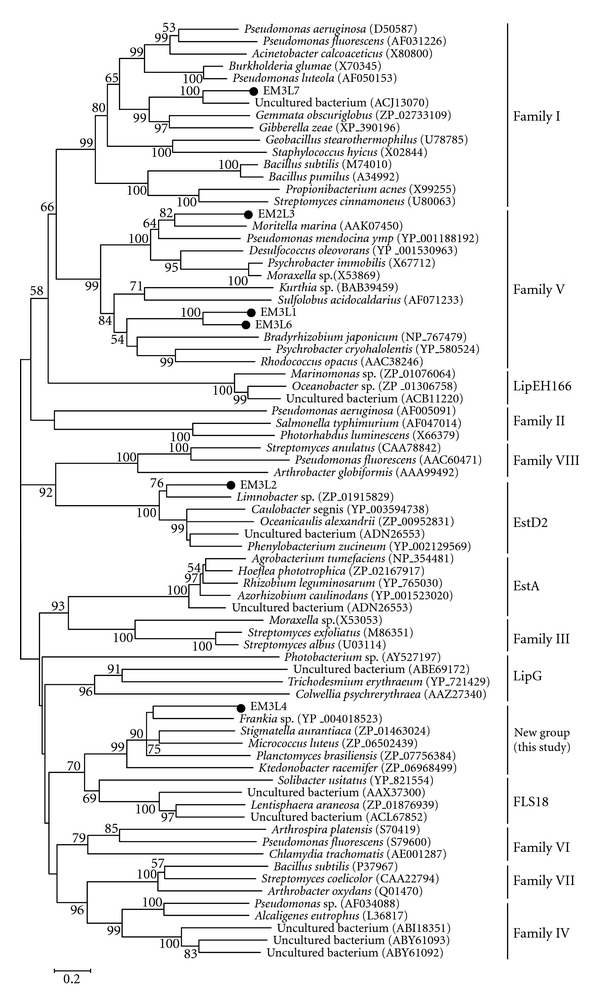
Phylogenetic tree of the lipolytic enzymes. The tree was constructed using the MEGA 4.1 program with the neighbor-joining algorithm. Only bootstrap values greater than 50% are shown. Bar: 0.2 substitutions per amino acid site.

**Figure 2:**
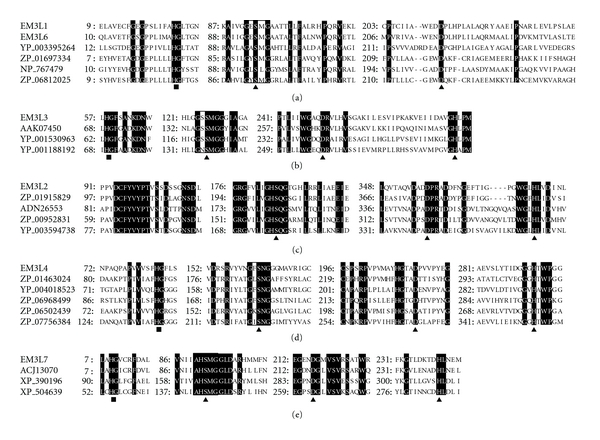
Multiple alignments of the conserved motifs of the ORFs isolated from metagenomic library with the homologs of each family or group. (a) Family V including EM3L1 and EM3L6: YP_003395264, an *α*/*β* hydrolase fold protein from *Conexibacter woesei* DSM 14684; ZP_01697334, an *α*/*β* hydrolase fold from *Bacillus coagulans* 36D1; NP_767479, a hypothetical protein bll0839 from *Bradyrhizobium japonicum* USDA 110; ZP_06812025, an *α*/*β* hydrolase fold protein from *Geobacillus thermoglucosidasius* C56-YS93. (b) Family V including EM3L3: AAK07450, a triacylglycerol acyl hydrolase from *Moritella marina*; YP_001530963, an *α*/*β* hydrolase fold from *Desulfococcus oleovorans *Hxd3; YP_001188192, an *α*/*β* hydrolase fold from *Pseudomonas mendocina* ymp (YP_001188192). (c) EstD2 family including EM3L2: ZP_01915829, a hypothetical protein from* Limnobacter* sp. MED105; ADN26553, an EstD2 from uncultured bacterium; ZP_00952831, a hypothetical protein from *Oceanicaulis alexandrii *HTCC2633; YP_003594738, a hypothetical protein from *Caulobacter segnis* ATCC 21756. (d) New group including EM3L4: ZP_01463024, an LpqC from *Stigmatella aurantiaca* DW4/3-1; YP_004018523, a lipoprotein from *Frankia* sp. EuI1c; ZP_06968499, a putative lipoprotein from *Ktedonobacter racemifer* DSM 44963; ZP_06502439, a conserved hypothetical protein from *Micrococcus luteus* SK58. (e) Family I including EM3L7: ACJ13070, a lipase from uncultured bacterium; (ACJ13070); XP_390196, a hypothetical protein from *Gibberella zeae* PH-1; XP_504639, a YALI0E31515p from *Yarrowia lipolytica*. Triangles and squares represent the residues involved in the formation of the catalytic triad and the oxyanion hole, respectively, and the conserved pentapeptide motifs are boxed.

To see how the ORFs were related to known esterases/lipases, the phylogenetic relationship was analyzed based on the esterase/lipase classification (family I–VIII) by Arpigny and Jaeger [1] including recently reported families such as LipG [35], EstA [36], LipEH166 [20], FLS18 [18], and EstD2 [17] (Figure 1). EM3L1, EM3L3, and EM3L6 were grouped into family V in Figure 1, *retaining* the G-X-S-M-G pentapeptide motif and the HG sequence for the oxyanion hole (Figures 2(a) and 2(b)). The His residue consisting of the catalytic triad could not be predicted in EM3L1 and EM3L6 by the sequence alignment (Figure 2(a)). EM3L2 was clustered together with EstD2 reported from metagenomic library of plant rhizosphere soil recently [17]. Multiple alignment analysis of EM3L2 revealed that the active site serine was encompassed by the conserved pentapeptide G-H-S-Q-G in EstD2 family (Figure 2(c)).

On the other hand, it was found that EM3L4 did not show significant similarity to any member in the eight families and branched out to a group together with close homologous genes. It is noteworthy that the comparative analysis of the deduced amino acid sequence revealed that EM3L4 displayed low similarity to poly(3-hydroxyalkanoate) depolymerase (ZP_07602911) from *Streptomyces violaceusniger* (34% identity), and both the active site serines in the two proteins were encompassed by G-X-S-N-G pentapeptide in common (Figure 2(d)). From the sequence analysis, it seems likely that EM3L4 could be a novel enzyme (Figure 1). Lastly, EM3L7 was clustered together with family I bacterial lipolytic enzymes, and the active site serine was encompassed by the characteristic A-X-S-X-G motif (Figure 2(e)). The motif was usually found in *Bacillus* lipases belonging to Subfamily I.4 even if no significant homology (less than 14% identity) was found between them. Taken together, the metagenomic study applied to marine sediment sample of deep sea has the potential to yield novel molecular entities unrelated to the known sequences.

### 3.2. Expression and Characterization of the Lipolytic Enzyme-Encoding Genes

**Figure 3:**
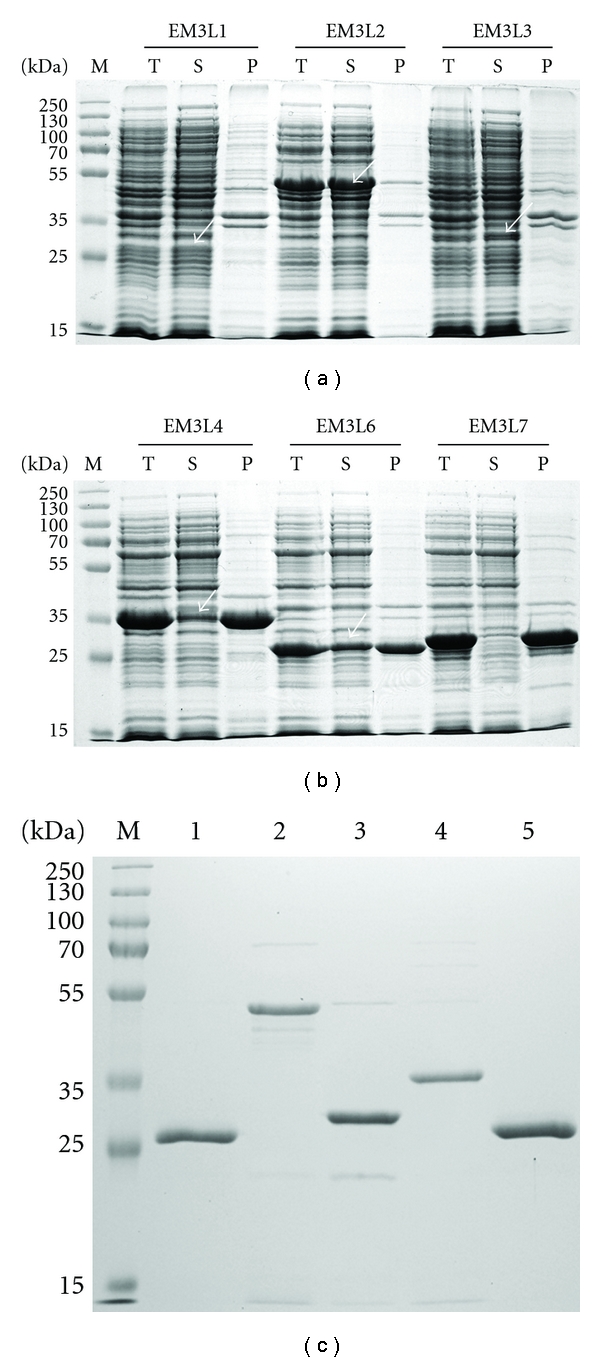
Expression of esterase/lipase genes. (a) Expression analysis of EM3L1, EM3L2, and EM3L3; (b) expression analysis of EM3L4, EM3L6, and EM3L7 with co-expression of the GroEL-GroES chaperone genes at 16°C; (c) purified enzymes. The bands corresponding to the proteins are indicated by arrows. Lane M: molecular mass standards, lane T: total cell lysate, lane S: soluble fraction, lane P: insoluble fraction, lane 1: purified EM3L1, lane 2: purified EM3L2, lane3: purified EM3L3, lane 4: purified EM3L4, and lane 5: purified EM3L6.

To express six genes, EM3L1, EM3L2, EM3L3, EM3L4, EM3L6, and EM3L7, we investigated the presence of signal sequences using the SignalP 3.0 program and found that EM3L2, EM3L3, and EM3L4 retained a putative signal sequence 18–25 amino acids long. All the genes with signal sequence removed were amplified, and the resulting expression constructs were expressed in *E. coli* BL21 strain. Total cell lysates, soluble fractions, and insoluble fractions were analyzed by 12% SDS-PAGE, and we found that some portion of the proteins encoded by three of the genes (EM3L1, EM3L2, and EM3L3) were detected in the soluble fraction (Figure 3(a)), whereas the proteins encoded by the three genes (EM3L4, EM3L6, and EM3L7) were expressed as insoluble forms.

Based on the fact that expression at low temperature increases the stability and proper folding of proteins, possibly due to the fact that the hydrophobic interactions that determine inclusion body formation are temperature dependent [37–39], we made three proteins (EM3L4, EM3L6, and EM3L7) being induced at the low temperature of 16°C. However, the induction of expression at low temperature alone was not successful in enhancing the solubility of the expressed proteins (data not shown). Since it has been reported that GroEL-GroES is effective in facilitating their correct folding by minimizing aggregation and misfolding [40], we employed GroEL-GroES co-expression in solubilizing the proteins. It turns out that EM3L4 and EM3L6 proteins were solubly expressed by coexpressing GroEL-GroES at 16°C. In contrast to the other proteins, the majority of the expressed EM3L7 remained in the insoluble fraction regardless of the experimental conditions we tried in this study (Figure 3(b)). The expressed proteins were purified as described in Section 2, and SDS-PAGE analysis of purified EM3L1, EM3L2, EM3L3, EM3L4, and EM3L6 showed a protein band which correlated well to the predicted molecular weight (Figure 3(c)). This result demonstrated that the combination of co-expression of chaperones and induction at low temperature may be effective in achieving soluble expression of lipolytic enzyme-encoding genes.

The purified enzymes showed an optimum activity in the temperature range of 30–35°C and at a neural or slightly alkaline pH (pH 7.5–8.5) (Table 2 and see Figures S1 and S2 in Supplementry Material available on line at doi:10.1155/2011/271419). It is noteworthy that they could be classified as cold-active enzymes based on the temperature profiles and activation energy values. As the source of the genes was a deep-sea sediment sample, we predicted that they are cold-adapted enzymes. A similar phenomenon has been reported in other studies; for example, two cold-adapted esterase from Arctic sediment metagenome showed an optimum temperature at 30°C [23] and thermostable esterase from hot spring metagenome showed enzyme activity above 30°C up to 95°C [41]. This result suggested that the property of lipolytic enzymes derived from metagenomic library might be reflected by their environmental characters.

EM3L1, EM3L2, EM3L3, and EM3L6 could hydrolyze short chain substrates (C_2_ to C_12_) and showed the highest activity toward *p*-Nitrophenyl hexanoate (C_6_) (Table 2 and Figure S3). On the other hand, EM3L4 showed enzymatic activity toward long-chain substrates (C_16_–C_18_), which can be classified as a lipase. The specific activities of the enzymes were determined to be in the range of 1.7–558.2 U/mg at the optimal conditions (Table 2). 

**Figure 4:**
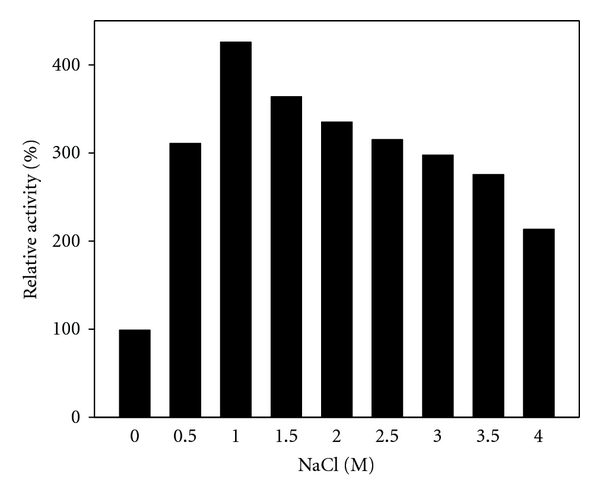
Effect of NaCl on *p*-nitrophenyl hexanoate hydrolysis activity of EM3L4. The activity of the enzyme preparation in the absence of NaCl before incubation was defined as the 100% level.

Since EM3L4 belonged to a new group and the homologous proteins have never been characterized, the biochemical property of EM3L4 was further analyzed. To determine the resistance to various chemical agents, the purified EM3L4 was incubated with chemical agents, and the remaining activity was measured with *p*-nitrophenyl palmitate or *p*-nitrophenyl hexanoate as substrate at 35°C. The activity of EM3L4 was increased by the presence of Mn^2+^, Mg^2+^, Ca^2+^, Cu^2+^, and Ni^2+^(Table 3). It was inhibited by Zn^2+^, Fe^2+^, and Co^2+^ and was completely inhibited by PMSF (Table 3). Besides, it was also inhibited by various nonionic detergents such as Tween-20, -40, -60, -80 and Triton X-100 and ionic detergent, SDS (Table 3). The enzyme activity of EM3L4 was significantly affected by salinity. As shown in Figure 4, EM3L4 displayed the highest enzyme activity in the presence of 1 M NaCl. Moreover, the activity was maintained with up to 4 M NaCl (Figure 4).

## 4. Discussion

Our approach to search for novel lipolytic enzymes started with the construction of metagenomic library from deep sea sediment. As a result, we could discover six lipolytic enzyme-encoding genes with low sequence similarity (33–58% identity) to the known proteins. By removing signal sequence in the N-terminus and co-expression of chaperone gene at low temperature, we could obtain soluble recombinant proteins encoded by 5 genes from *E. coli*. The biochemical characterization of them revealed being cold-adapted, reflecting the environmental feature of the origin of metagenome. 

Particularly, EM3L4 branched out to a new group in the phylogenetic tree, and any of homologous proteins to EM3L4 has never been characterized. The purified EM3L4 preferred to hydrolyze longer fatty acids, and highly active at high NaCl concentration. Recently, some esterases whose activities were increased in the presence of NaCl have been identified using metagenomic libraries of various marine environments such as deep-sea water [42], sea water [36], and sea sediment [18]. We suggest that EM3L4 is a novel lipase retaining salt-resistance property. Then, a question arises whether homologous proteins belonging to the same group with EM3L4 show similar properties, and it will need further investigation. 

Conclusively, this study demonstrated that novel lipolytic enzymes in terms of primary sequence, activity profile, and substrate specificity could be obtained by metagenomic approach using deep-sea sediment sample. They could be potentially used as a biocatalyst in pharmaceutical and fine chemical industries.

## Figures and Tables

**Table 1:** Primers used in the cloning of lipolytic enzyme-encoding genes.

Gene	Primer*
EM3L1	5′-TTTGGAGGCATATGCTTATCCCTTCCGATGGTCTGGAAC-3′
	5′-CTGGTGTCCTCGAGGTTTGCCAAAAAACGGCCGTATATC-3′
EM3L2	5′-TGATAAATCATATGAACAGAATGACAATAGGCTTCTC-3′
	5′-CCTTTTAGCTCGAGATTATTGTCTTGCAACCAGGATT- 3′
EM3L3	5′-TAGCTGCACATATGATCCTTTTTAGCTTGGTCGGTGTC-3′
	5′-AAAGTTGGCTCGAGTGTCTTAATCCCAAGAAAATTCAAG-3′
EM3L4	5′-GAGAAGTGAACCGGGGGACATATGACTGGTAGAATTG-3′
	5′-CAAAAGCGCCCCTCGAGCGGCTGCTCCTCC-3′
EM3L6	5′-AGGAGAAGCATATGAAATGCATCCCATCAGACGGCC-3′
	5′-GTTGCAGACTCGAGATCATTATTCGAGCCTAATTCCTC-3′
EM3L7	5′-CGGAGTGAAAGCATATGACTTATCCGATTGTGCTC-3′
	5′-GTATTACCTCTCGAGGTTAAGGTAGTTGTTCCGCGATTC-3′

*Underlined bases in the primers indicate the restriction enzyme recognition site (NdeI/XhoI).

**Table 2:** Characteristics of the purified lipolytic enzymes.

Enzyme	Temperature^a^ (optimum) (°C)	Activation energy (kcal/mol)	pH^b^ (optimum)	Substrate^c^ (optimum)	Specific activity (U/mg)
EM3L1	15–50 (35)	3.6	7.5–9.5 (8.5)	C_2_–C_10_ (C_6_)	558.2
EM3L2	15–40 (30)	8.6	7.0–10.0 (8.5)	C_6_ (C_6_)	1.7
EM3L3	15–40 (35)	3.5	7.0–10.0 (8.5)	C_2_–C_10_ (C_6_)	29.7
EM3L4	20–50 (35)	10.6	7.0–10.0 (7.5)	C_16_–C_18_ (C_16_)	5.3
EM3L6	15–40 (35)	4.1	7.5–9.0 (8.5)	C_2_–C_10_ (C_6_)	23.3

^a^The temperature range is the temperatures at which the activities are greater than 50% of the highest value (Figure S1). ^b^pH range is the pHs at which the activities are greater than 50% of the highest value (Figure S2). ^c^Substrates are the *p*-nitrophenyl esters for which the enzyme shows an activity greater than 20% of the highest value (Figure S3).

**Table 3:** Effect of metal ions and detergents on EM3L4.

Metal ions (1 mM)	Relative activity (%)	Detergent (1%)	Relative activity (%)
None	100	None	100
MnCl_2_	147	Tween 20	74
MgCl_2_	175	Tween 40	70
CaCl_2_	175	Tween 60	54
CuCl_2_	165	Tween 80	69
ZnSO_4_	52	Triton X-100	92
FeSO_4_	0	SDS	0
CoSO_4_	35		
NiSO_4_	112		
PMSF	42		
EDTA	107		
